# Coupling coordination relationship between health resource allocation and regional economic development: an empirical study based on five provinces in eastern China

**DOI:** 10.3389/fpubh.2024.1513188

**Published:** 2024-12-16

**Authors:** Yongqiang Wang, Xiaochen Feng, Yulin Chai, Kexuan Chen, Shilan Yang, Wei Li, Yuqing Mi

**Affiliations:** ^1^School of Management, Shandong Second Medical University, Weifang, China; ^2^School of Public Health, Shandong Second Medical University, Weifang, China

**Keywords:** health resource allocation, economic development, coupling coordination degree, fixed effects model, high-quality development

## Abstract

**Background:**

Improving system coordination is a pivotal strategy and a critical pathway for social governance. Chinese society is currently facing a significant challenge in aligning the allocation of health resources with economic development. Evaluating the level of coordinated development within the system can provide valuable insights to support the construction of a more coordinated China and foster high-quality development.

**Methods:**

Based on a systematically constructed indicator framework, our study selected data from five eastern provinces of China to establish a ten-year panel dataset covering the period from 2011 to 2020. The comprehensive evaluation index and the relative development degree were employed to comprehensively evaluate the development level of the system. The coupling coordination degree model was applied to analyze the coupling coordination relationship and spatiotemporal evolution trend of the two systems. Additionally, the fixed effects model was used to identify the driving factors behind the coordinated development of the two systems.

**Results:**

From 2011 to 2020, the comprehensive indices of health resource allocation and economic development in the five eastern provinces of China exhibited a consistent year-on-year increase, and the relative development degree experienced two critical values of 0.8 and 1.2, which changed from the lagging allocation of health resources to the lagging economic development. The system coordination index generally ranged between 0.35 and 0.90, with the coordination phase undergoing a transition from an antagonistic stage to a coordinated stage. The coordination type also gradually shifted from mild imbalance to good coordination. Furthermore, the levels of economic development, economic structure, technological investment, as well as the allocation of health human and material resources, all serve as critical drivers in enhancing the coordinated development of the system.

**Conclusion:**

The coordinated development of eastern China’s provinces produces substantial spillover effects, and the realization of a Healthy China initiative must strategically harness their radiative and demonstrative effects. Achieving a superior level of coordination requires urgent efforts to rectify the existing deficiencies in the distribution of grassroots healthcare resources. Furthermore, cultivating innovative drivers of economic growth and enhancing the capacity for economic support are critical to ensuring high-quality and sustainable development.

## 1 Introduction

The relationship between healthcare and economic development has long been a subject of interest for policymakers and researchers alike. A large number of empirical studies have demonstrated that healthcare and economic development are interactive (1–4). On the one hand, economic development serves as both a prerequisite and a catalyst for advancements in healthcare (5). The level of economic development determines the financial resources allocated to healthcare, with higher economic prosperity typically leading to increased investment in health services (6). On the other hand, the development of healthcare establishes a solid foundation for economic growth (7). The population with access to healthcare services transforms into a healthy workforce. This workforce increases the country’s productivity, thereby contributing to economic development (8). Health resource allocation, as the means and guarantee for the development of healthcare, plays a critical role in enhancing the efficiency of healthcare services and promoting health equity (9, 10). For instance, studies have demonstrated that appropriate distribution of primary healthcare resources can reduce avoidable hospitalizations related to diabetes (11). Additionally, improvements in the spatial accessibility of healthcare facilities can significantly boost service efficiency and promote equitable health outcomes (12). The interaction between them has injected a strong impetus into the stable development of society. Although the objectives of health resource allocation and economic development may differ, they share fundamental similarities in that both rely on the rational distribution and utilization of human, financial, and material resources (13, 14). As such, exploring the synergistic relationship between these two systems is of paramount importance for optimizing health resource allocation and further advancing economic growth.

The relationship between health and the economy has had a profound and enlightening impact on China (15). According to the government’s annual report, the past decade has witnessed the fastest economic growth in China’s history, alongside significant advancements in healthcare. However, in the context of the 14th Five-Year Plan, Chinese society faces the challenge of transitioning from high-speed growth to high-quality development (16). In terms of economic development, China is grappling with a severe supply–demand imbalance, and the imperative for high-quality economic development necessitates both a transformation in growth momentum and structural adjustments (17). In the health sector, challenges such as resource redundancy, misallocation, and inefficiency persist, with issues of equity and efficiency in health resource distribution requiring urgent improvement (18). Given the bidirectional causal relationship between health and economic development, addressing these issues demands a solution grounded in system coordination. Thus, achieving coordinated development between health and the economy has become a critical issue for resolving systemic development challenges in the new era.

In October 2016, the *Healthy China 2030 Plan Outline* introduced the principle of prioritizing health, emphasizing innovation, coordination, sustainability, openness, and shared development as guiding concepts to steer both the healthcare sector and economic growth (19). The Report of the 20th National Congress of the Communist Party of China further underscored that system coordination is an essential requirement for achieving high-quality development in the new era (20). These policy initiatives have elevated the coordinated development of healthcare and the economy to a national strategic priority, reflecting the significant importance placed by Chinese society on aligning the allocation of health resources with economic growth. It is increasingly recognized that optimal outcomes are achieved when health and economic systems evolve synergistically, interactively, and in a synchronized manner (21). Our research orientation is in line with the needs of current social development in China. To more effectively explore the coordination between health resource allocation and high-quality economic development, we selected Shandong, Jiangsu, Zhejiang, Fujian, and Guangdong provinces for our study. These provinces, characterized by similar geographical settings, high population density, and advanced levels of economic and healthcare development, provide a representative context to identify realistic pathways for achieving high-quality development in China.

According to existing research, we found that most studies on the coupling coordination relationship focus on economic and environmental systems, Hou et al. (22). explored the coupling and coordination of China’s economy, ecological environment, and public health from a green production perspective. Similarly, Zhang et al. (23). examined the coupling and coordination between green finance, the digital economy, and the ecological environment in China. These studies primarily aim to discuss the coordinated development of complex systems. In the context of systemic coordination between health resource allocation and economic development, Liu et al. (24). explored the coordinated relationship between health resource allocation and economic development in China from a holistic perspective. Tang et al. (25). analyzed the spatial–temporal characteristics and driving forces behind the coordinated development of these two systems. Moreover, other studies have assessed the level of coordinated development from a provincial standpoint (26–28). Building upon this body of work, we employ the coupling coordination degree model to assess the development level and coordination status of the systems in the five provinces of East China. Furthermore, we apply the fixed effects model to examine the driving forces behind the high level of system coordination in these regions. This approach allows for a deeper understanding of the factors influencing the synergistic relationship between health resource allocation and economic development.

The innovations and contributions of this paper are as follows: (1) We selected specific provinces for evaluation based on their unique characteristics, and the findings offer valuable insights for the coordinated development of Chinese society in the context of high-quality development. (2) This study further enriches the understanding of the relationship between healthcare and economic development, providing a case study from China that can serve as a reference for countries and regions with comparable levels of healthcare and economic development in managing the interplay between health resource allocation and economic growth. (3) The study examines the spatiotemporal evolution and driving factors of the coupling and coordination relationship from a systems-theoretic perspective, offering a reference for the development of China’s central and western regions.

## 2 Methods

### 2.1 Selection of indicators

Guided by the principles of systematicity, comparability, and data accessibility, we constructed a system of indicators for evaluating health resource allocation and economic development. For health resource allocation, we considered not only the allocation of financial, material, and human resources within the healthcare sector, but also constructed evaluation indicators based on total health resource allocation, *per capita* health resource allocation, and health resource allocation per unit of land area. Regarding economic development, we formulated indicators from the perspectives of the economic scale, structure, dynamics, and efficiency, which systematically evaluate production, investment, consumption, openness, income, price changes, and industrial structure. All the indicators we used for this study are visible in Table 1.

**Table 1:** Evaluation Indicator system of coordinated development of health resource allocation and economy.

Target Layer	System Layer	System Layer	Indicator Layer	Indicator Direction
Allocation Level of Health resource	Aggregate allocation level	Total financial resource	Total health expenditure	+
		Total material resource	Total number of medical institutions	+
			Total number of beds in medical institutions	+
		Total human resource	Total number of practicing (assistant) physicians	+
			Total number of registered nurses	+
	*Per capita* allocation level	*Per capita* financial resource	*Per capita* health expenditure	+
		*Per capita* material resource	Number of medical institutions per 1,000 population	+
			Number of beds in medical institutions per 1,000 population	+
		*Per capita* human resource	Number of practicing (assistant) physicians per 1,000 population	+
			Registered nurses per 1,000 population	+
	Per land allocation level	Distribution of material resource	The distribution density of medical institutions	+
			The distribution density of beds in medical institutions	+
		Distribution of human resource	The distribution density of practicing (assistant) physicians	+
			The distribution density of registered nurses	+
Economic Development Level	Economic Scale	Productivity Level	GDP	+
		Consumption Level	Total retail sales of social consumer goods	+
		Trade Level	The total volume of imports and exports	+
	Economic Structure	Industrial Structure	Proportion of secondary industry to GDP	+
			Proportion of tertiary industry to GDP	+
		Technical Structure	Proportion of technology market turnover to GDP	+
	Economic Dynamics	Investment Momentum	The growth rate of fixed-asset investment	+
			Growth rate of general public budget expenditure	+
		Consumption Momentum	Total retail sales of consumer goods *per capita*	+
		Trade Momentum	The growth rate of exports and imports	+
		Innovation Momentum	Number of patents granted	+
	Economic Benefits	Resident Income	*Per capita* disposable income	+
		Price Fluctuation	CPI	−
		Employment Level	Urban registered unemployed population.	−

“+” indicates the positive indicators, and “−” indicates the negative indicators.

### 2.2 Data sources

We selected the statistics related to health resource allocation and the economic development of five provinces along the eastern coast of China from 2011 to 2020 as the analysis object for our study, which was obtained from the China Statistical Yearbook, the China Health Statistical Yearbook, the China Health and Family Planning Statistical Yearbook, the statistical yearbooks of each province and the National Economic and Social Development Statistical Bulletin from 2011 to 2022.

### 2.3 Research methodology

#### 2.3.1 Comprehensive evaluation index

The system of indicators for evaluating health resource allocation and economic development has different dimensions, and each indicator has a different influence on the system. To ensure the scientificity and objectivity of the evaluation results, the study first adopted the entropy assignment method to determine the weight of all indicators (29), based on which the study adopted the linear weighting method to calculate the comprehensive evaluation index of the health resource allocation system and the economic development system (30). The comprehensive evaluation index is a quantitative index reflecting the comprehensive development level of the system (31). The formula is as follows:


(1) $$U=\overset{m}{\underset{j-1}{\sum }}w_{j}/X{'}_{ij}\text{.}$$

Where *U* is the comprehensive evaluation index of the system, *ω_j_* is the weight of the evaluation indicator of item *j*, and *X’_ij_* is the standardized value of each indicator.

#### 2.3.2 Relative development degree

Based on the comprehensive evaluation of the system, our study utilized the relative development degree to measure the relative development level of health resource allocation and economic development. The formula is as follows:


(2) $$S=U_{h}/U_{e}\text{.}$$

Where *S* denotes the relative development degree, *U_h_* is the comprehensive evaluation index of the health resource allocation system, and *U_e_* is the comprehensive evaluation index of the economic development system. According to the existing research, *S* ≥ 1.2 indicates that the level of health resource allocation is better than the level of economic development, and economic development is relatively lagging; 0.8 < *S* < 1.2 indicates that health resource allocation and economic development are in a state of dynamic equilibrium, and there is a good interaction between systems, and they are mutually promoting; *S* ≤ 0.8 indicates that the level of health resource allocation is lagging behind the level of economic development, which requires further increase in health resource input or optimization of health resource allocation to adapt and support economic development (32).

#### 2.3.3 Coupling coordination degree model

The Coupling Coordination Degree Model (CCDM) is a widely used approach to study how different subsystems interact and evolve in a coordinated manner (33). The CCDM is built based on the theoretical concepts of coupling and coordination. Coupling indicates the degree of interaction and mutual influence of two or more systems, which is often described by the degree of coupling in research (34). Coordination represents the level of coordinated development of two systems, which is reflected by the system coordination index (35). The CCDM is an evaluation model based on these two concepts. Therefore, we constructed the CCDM of health resource allocation and economic development to explore the level of coordinated development of these two systems. The equations are as follows:


(3) $$C=2\times \sqrt{\left[\frac{U_{h}\times U_{e}}{\left(U_{h}+U_{e}\right)\left(U_{h}+U_{e}\right)}\right]}\text{.}$$

(4) $$T=\alpha U_{h}+\beta U_{e}\text{.}$$

(5) $$D=\sqrt{C\times T}\text{.}$$

Where *C* is the degree of coupling, and the range of values is [0, 1]. The higher the value of *C* is, the greater the degree of mutual influence of the two systems. *T* is the system coordination index, and *α* and *β* are coefficients to be determined. After reviewing a large amount of relevant information, we found that the degree of interaction between health resource allocation and economic development has not yet been determined by academics (24). Therefore, in our study, health resource allocation and economic development are considered equally important, and both *α* and *β* were set to 0.5 for the empirical study. Where *D* is the coupling coordination degree, *D* ranges from 0 to 1, and the closer the value of *D* is to 1, the better the relationship between health resource allocation and economic development. The coupled coordination stages, coupled coordination types, and coupled coordination grades corresponding to the *D* values are shown in Table 2.

**Table 2:** Criteria for determining the coupling coordination degree between health resource allocation and economic development.

Coupling coordination stage	Range of *D* value	Coupling coordination type	Coupling coordination grade
Low-level coordination (antagonistic period)	0 ≤ *D* < 0.1	Extreme disorder	I
	0.1 ≤ *D* < 0.2	Severe disorder	II
	0.2 ≤ *D* < 0.3	Moderate disorder	III
	0.3 ≤ *D* < 0.4	Mild disorder	IV
Medium-level coordination (breaking-in period)	0.4 ≤ *D* < 0.5	Endangered disorder	V
	0.5 ≤ *D* < 0.6	Some coordination	VI
High-level coordination (coordination period)	0.6 ≤ *D* < 0.7	Primary coordination	VII
	0.7 ≤ *D* < 0.8	Moderate coordination	VII
	0.8 ≤ *D* < 0.9	Good coordination	IX
	0.9 ≤ *D* < 1.0	High-quality coordination	X

#### 2.3.4 Fixed effects model

The data used in our study are the panel data of five provinces in eastern China from 2011 to 2020. In order to take full advantage of the information provided by the panel data and to avoid endogenous problems caused by omitted explanatory variables, we chose the Fixed Effects Model (FEM) to explore the driving factors influencing the coupling coordination degree (36). Regarding the variable selection, we take the coupling coordination degree as the dependent variable. The explanatory variables are chosen as follows: *per capita* GDP (*x*_1_) to measure regional economic development, the proportion of secondary and tertiary industry output in GDP (*x*_2_) to measure industrial structure, the proportion of the budget expenditure of science and technology in fiscal expenditure (*x*_3_) to measure the investment in science and technology, *Per capita* health expenditure (*x*_4_) as the indicator of financial resources invested in health, the proportion of practicing (assistant) physicians and registered nurses in health technicians (*x*_5_) to measure human resources investment, and the number of sickbeds per 1,000 residents (*x*_6_) to measure the material resources investment in health. The expansion model was set as follows:


(6) $$\begin{array}{l} \ln D_{\mathrm{it}}=\alpha +\beta \ln x_{1,\mathrm{it}}+\beta \ln x_{2,\mathrm{it}}+\beta \ln x_{3,\mathrm{it}}+\beta \ln x_{4,\mathrm{it}}+\\ \beta \ln x_{5,\mathrm{it}}+\beta \ln x_{6,\mathrm{it}}+\delta_{i}+\eta_{t}+\varepsilon_{\mathrm{it}}\text{.} \end{array}$$

Where *D_it_* indicates the coupling coordination degree between health resource allocation and economic development, *i* indicates region, *t* indicates time, *α* is a constant term, *β* is the coefficient of explanatory variables, *δ_i_* denotes individual effects across provinces, *η_t_* denotes time effects across years, and *ε_it_* indicates the random noise terms (37, 38). The validity of the Fixed Effects Model (FEM) constructed in this study depends on several key assumptions, namely the zero conditional mean assumption of the error term, the homoscedasticity assumption, the absence of serial correlation, and the independence of the fixed effects (39). The zero conditional mean assumption defines the expectation of *ε_it_* as zero, with no correlation between the error term and each explanatory variable:


(7) $$E\left(\varepsilon_{\mathrm{it}}|\ln x_{\mathrm{it}}\right)=0$$

The homoscedasticity assumption defines the variance of the error term as constant, with no dependence on any of the explanatory variables:

(8) $$Var\left(\varepsilon_{\mathrm{it}}|\ln x_{\mathrm{it}}\right)=\sigma^{2}$$.

The absence of serial correlation assumption defines that there is no serial correlation between the error terms, meaning that:

(9) $$Cov\left(\varepsilon_{\mathrm{it}},\;\varepsilon_{js}\right)=0\;\left(for\;i\neq \;j\;\mathrm{or}\;t\neq \;s\right)$$.

The independence assumption of the fixed effects defines that both *δ_i_* and *η_t_* are independent of the explanatory variables. This is a key assumption for the validity of the Fixed Effects Model (FEM):

(10) $$E\left(\delta_{i}|\ln x_{\mathrm{it}}\right)=0$$.

(11) $$E\left(\eta_{t}|\ln x_{\mathrm{it}}\right)=0$$.

In summary, the FEM constructed in this study provides a robust econometric tool for investigating the driving factors behind the coupling coordination degree between health resource allocation and economic development. Furthermore, the verification and correction of the aforementioned assumptions ensure the robustness and validity of the model’s results.

## 3 Results

### 3.1 Systematic evaluation of health resource allocation and regional economic development

According to the results of the comprehensive evaluation of systems, the comprehensive evaluation index of the health resource allocation and economic development of the five eastern provinces of China was increasing year by year. From 2011 to 2020, the allocation of health resources was rapidly optimized, and the level of economic development maintained stability and progress. From the comparative analysis of the results of the comprehensive evaluation of health resource allocation and economic high-quality development in five eastern provinces (Table 3), from 2011 to 2013, the different values of the comprehensive evaluation index of Shandong Province, Jiangsu Province, Zhejiang Province, Fujian Province, and Guangdong Province were all negative, and the relative development degree of these two systems were all less than 0.8. This suggests that, during this period, the allocation of health resources lagged behind economic development, highlighting the need for increased and continuous optimization of health resource distribution. Between 2014 and 2015, while some provinces still recorded negative differences in the comprehensive evaluation index, the relative development index of the two systems ranged between 0.8 and 1.2. This indicates that the allocation of health resources had been optimized, with health resource allocation and economic development approaching a dynamic balance. From 2016 to 2020, the difference values of the comprehensive evaluation index for all five provinces were positive, with relative development index values exceeding 0.8 in each case. Notably, in some provinces, the relative development index exceeded 1.2, suggesting that, after reaching an initial balance, the allocation of health resources outpaced economic development.

**Table 3:** Comparative analysis of comprehensive evaluation results between health resource allocation and economic development.

Year	Shandong Province		Jiangsu Province		Zhejiang Province		Fujian Province		Guangdong Province	
	*U_h_-U_e_*	*S*	*U_h_-U_e_*	*S*	*U_h_-U_e_*	*S*	*U_h_-U_e_*	*S*	*U_h_-U_e_*	*S*
2011	−0.150	0.281	−0.122	0.382	−0.194	0.299	−0.147	0.428	−0.176	0.382
2012	−0.226	0.333	−0.162	0.414	−0.132	0.447	−0.189	0.461	−0.112	0.606
2013	−0.037	0.704	−0.104	0.664	−0.141	0.467	−0.129	0.692	−0.104	0.737
2014	0.032	1.087	0.011	1.033	−0.088	0.806	−0.001	0.997	−0.008	0.983
2015	0.027	1.064	0.039	1.112	0.000	0.999	−0.010	0.979	0.043	1.081
2016	0.028	1.052	0.070	1.163	0.013	1.031	0.063	1.123	0.100	1.184
2017	0.025	1.043	0.070	1.125	0.101	1.221	0.109	1.198	0.124	1.208
2018	0.143	1.237	0.058	1.093	0.136	1.242	0.154	1.249	0.159	1.257
2019	0.180	1.286	0.077	1.114	0.104	1.151	0.177	1.270	0.195	1.308
2020	0.185	1.259	0.152	1.207	0.118	1.160	0.173	1.241	0.215	1.319

### 3.2 Spatial and temporal evolution of the coupling coordination relationship between health resource allocation and economic development

**Figure 1:**
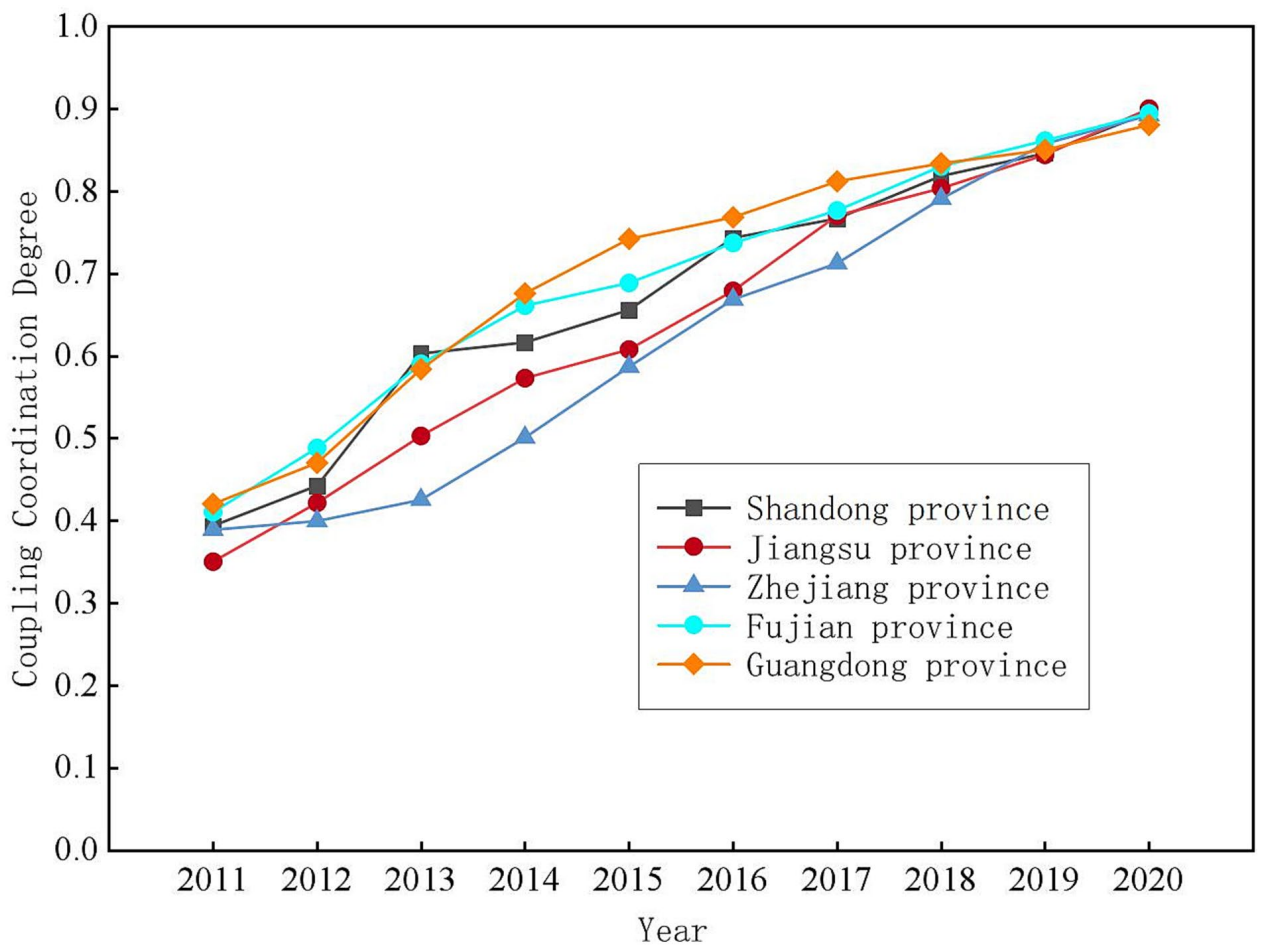
Evolutionary trends of the coupling coordination degree between health resource allocation and economic development from 2011 to 2020.

**Figure 2:**
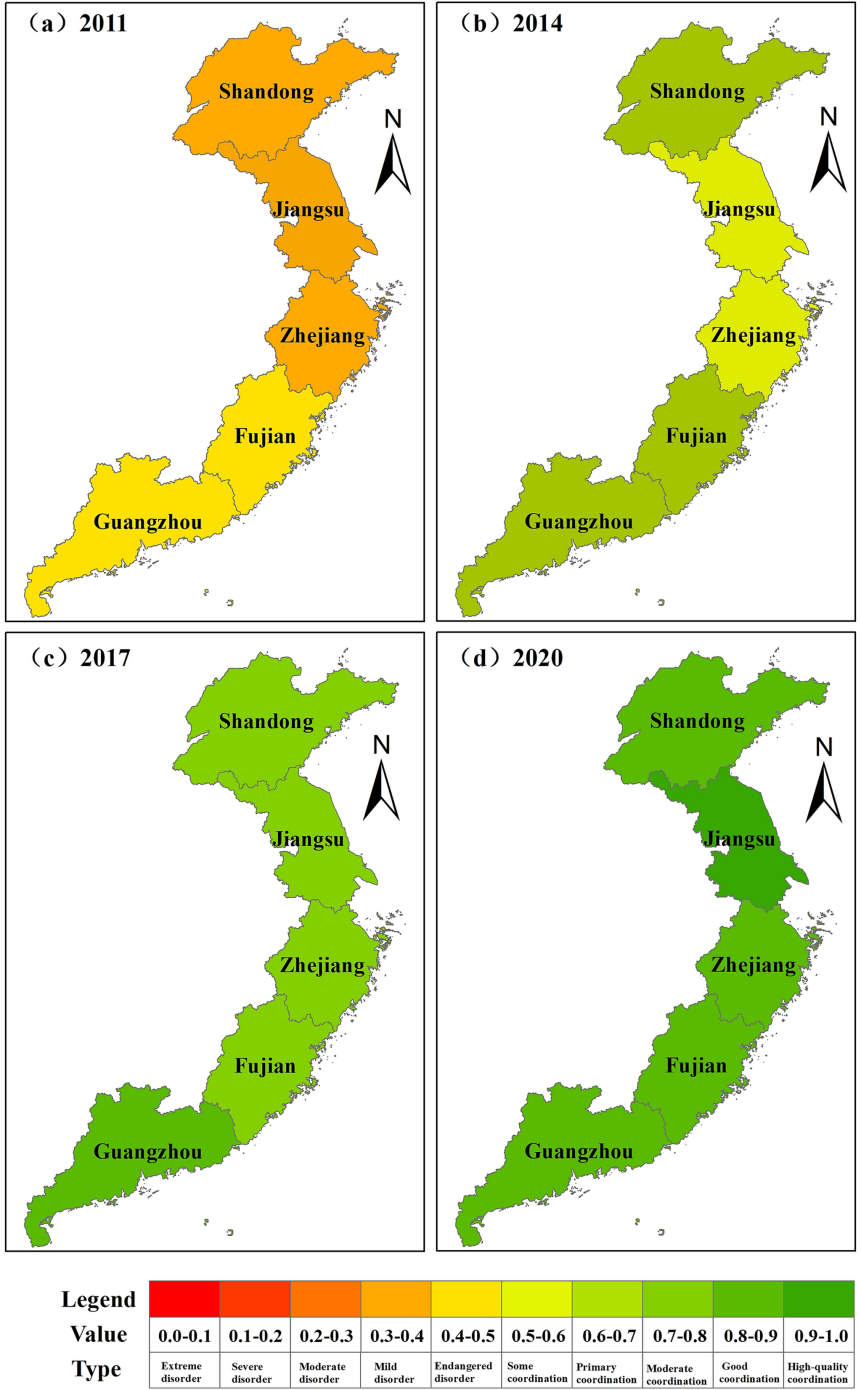
Spatial distribution characteristics of the coupling coordination type between health resource allocation and economic development from 2011 to 2020.

From the perspective of temporal evolution and spatial evolution, from 2011 to 2020, the coupling coordination relationship between health resource allocation and economic development in the five provinces of eastern China went through three stages: low-level coordination (antagonism period), medium-level coordination (breaking-in period), and high-level coordination (coordination period). The coupling coordination degree was generally between [0.35, 0.90]. The coupling coordination relationship has been optimized year by year. As seen in Figure 1, from 2011 to 2012, the coupling coordination relationship between health resource allocation and economic development in all provinces was mildly imbalanced or endangered. As seen in Figure 2, the type of coupling coordination relationship in Shandong, Zhejiang, and Jiangsu provinces was mildly imbalanced in 2011, and the coupling coordination degree of the three provinces was 0.3936, 0.3887, and 0.3499, respectively. The coupling coordination degree of Guangdong Province and Fujian Province was relatively high, and the coupling coordination degree was 0.4101 and 0.4199, respectively. The type of coupling coordination relationship was an endangered imbalance. According to the coupling coordination degree, we can see that the health resource allocation and economic development of the provinces in eastern China were in the low-level coupling coordination stage. In Figure 1, we found that the coupling coordination relationship between health resource allocation and economic development in all provinces changed from medium-level coordination to high-level coordination from 2012 to 2017. The type of coupling coordination relationship also moved from endangered imbalance to moderate coordination. From the inclination of the line in Figure 1, we judged that the coupling coordination relationship between health resource allocation and economic development developed the fastest in this period. The change is also evident in the type of coupling and coordination between the provinces from Figure 2. From 2017 to 2019, the coupling coordination degree of health resource allocation and economic development in all provinces was greater than 0.7, and the coupling coordination relationship belonged to moderate coordination and good coordination. In 2020, the allocation of health resources and economic development in Jiangsu Province reached high-quality coordination (*D* = 0.9001), and the rest of the provinces were close to high-quality coordination. The types of coupling coordination relationships in 2000 are shown in Figure 2.

### 3.3 Analysis of the drivers affecting the allocation of health resources in harmony with economic development

#### 3.3.1 Model building result

To avoid the pseudo regression problem, the study first adopted the *LLC* test, *IPS* test, and *Fisher-PP* test to carry out the unit root test on the time series. The test results are shown in Table 4. From the test results, we found that the *p* value of each variable series after first-order differencing is less than 0.05 at the 5% significance level. Therefore, the variable series passes the unit root test. This indicates that the stability of the time series after first-order differencing is good and meets the needs of the model.

**Table 4:** Results of the LLC test, IPS test, and Fisher-PP test.

Variable	Test method	*P* value	Stationarity	First-order difference variable	Test method	*P* value	Stationarity
Ln*D*	*LLC*	<0.001	Stationary	*D*Ln*D*	*LLC*	<0.001	Stationary
	*IPS*	0.016 9	Stationary		*IPS*	0.005 4	Stationary
	*Fisher-PP*	<0.001	Stationary		*Fisher-PP*	0.025 7	Stationary
Ln*x*_1_	*LLC*	<0.001	Stationary	*D*Ln*x*_1_	*LLC*	<0.001	Stationary
	*IPS*	0.309 7	Nonstationary		*IPS*	0.013 2	Stationary
	*Fisher-PP*	0.002 7	Stationary		*Fisher-PP*	<0.001	Stationary
Ln*x*_2_	*LLC*	<0.001	Stationary	*D*Ln*x*_2_	*LLC*	<0.001	Stationary
	*IPS*	0.104 5	Nonstationary		*IPS*	0.003 5	Stationary
	*Fisher-PP*	0.961 8	Nonstationary		*Fisher-PP*	0.014 8	Stationary
Ln*x*_3_	*LLC*	0.003 5	Stationary	*D*Ln*x*_3_	*LLC*	<0.001	Stationary
	*IPS*	0.245 6	Nonstationary		*IPS*	0.002 1	Stationary
	*Fisher-PP*	0.815 3	Nonstationary		*Fisher-PP*	0.001 1	Stationary
Ln*x*_4_	*LLC*	<0.001	Stationary	*D*Ln*x*_4_	*LLC*	<0.001	Stationary
	*IPS*	0.024 5	Nonstationary		*IPS*	<0.001	Stationary
	*Fisher-PP*	<0.001	Stationary		*Fisher-PP*	<0.001	Stationary
Ln*x*_5_	*LLC*	<0.001	Stationary	*D*Ln*x*_5_	*LLC*	<0.001	Stationary
	*IPS*	0.002 7	Stationary		*IPS*	<0.001	Stationary
	*Fisher-PP*	0.157 1	Nonstationary		*Fisher-PP*	<0.001	Stationary
Ln*x*_6_	*LLC*	<0.001	Stationary	*D*Ln*x*_6_	*LLC*	<0.001	Stationary
	*IPS*	0.043 5	Nonstationary		*IPS*	0.025 4	Stationary
	*Fisher-PP*	<0.001	Stationary		*Fisher-PP*	<0.001	Stationary

The *Pedroni* test and *Westerlund* test were applied to test the cointegration of the variable series, and the results are shown in Table 5. We found that the *p* value is less than 0.05 at the 5% level of significance, and the test results indicate that there is a long-term and stable relationship between the selected variables.

**Table 5:** Results of Pedroni test and Westerlund test.

Test method	Statistic	Statistical value	*P* value
Pedroni test	*Modified Phillips-Perron t*	4.034 7	<0.001
	*Phillips-Perron t*	−12.426 3	<0.001
	*Augmented Dickey-Fuller t*	−9.430 6	<0.001
Westerlund test	*Variance ratio*	4.209 1	<0.001

Finally, we determined the specific model category through the *F* test, *LM* test, and modified *Hausman* test, as shown in Table 6. The *p* value of the *F* test is less than 0.05 at the 5% significance level. The *p* value of the *LM* test is greater than 0.05 at the 5% significance level. The *p* value of the modified Hausman test is also less than 0.05 at the 5% significance level. The results of the above tests rejected the original hypotheses of the Mixed Effects Model and the Random Effects Model, so we chose the FEM for regression analysis of panel data.

**Table 6:** Results of the *F* test, *LM* test, and modified Hausman test.

Test method	Statistic	Statistical value	*P* value
*F* test	*F*	59.36	<0.001
*LM* test	*chibar2*	0.00	1.000 0
Modified Hausman test	*chi2*	36.93	<0.001

#### 3.3.2 Analysis of regression results

The regression results of the FEM (Table 7) show that the coordinated development of health resource allocation and economy in the five eastern provinces is significantly affected positively by the level of economic development, industrial structure, scientific and technological investment, and health human resources and health facility staffing. In terms of the specific degree of influence, the regression coefficient of *per capita* GDP is 0.247 8, with the greatest influence on the coordinated development of the systems in the five eastern provinces, which indicates that the level of economic development is the primary driving force for coordinated development and plays a supportive role in promoting coordinated development between health resource allocation and economic development. The regression coefficients of health human resources and health facility resource investment are 0.183 8 and 0.122 3, both of which have an important influence on the coordinated development of the systems. This influence is specifically explained by the fact that the inputs of health human resources and health material resources are transformed into factors promoting economic development, which have a simultaneous driving effect on the high-quality development of the economy and the progress of the health industry, which further promotes the system to achieve higher coordination. The regression coefficient of the relevant index of industrial structure is 0.072. This demonstrates that the rational optimization of industrial structure can generate the internal driving force for the coordinated development of health resource allocation and economy, and with the expansion of the proportion of the service industry in the economic structure, as a specific form of the service industry, medical and health services also have an important impact on the coordinated development of health resource allocation and economy. Scientific and technological investment, as the most promising driver of economic development, is increasingly integrated into health care services. The regression results show that the degree of influence of scientific and technological investment on the coordinated development of health resource allocation and economic development is 0.021.

**Table 7:** The regression results of FEM.

Variable	Coefficient	*t* value	*P* value
Ln*x*_1_	0.247 8^***^	7.07	<0.001
Ln*x*_2_	0.072^*^	2.41	0.020
Ln*x*_3_	0.021^*^	2.07	0.045
Ln*x*_4_	0.164	0.55	0.585
Ln*x*_5_	0.183 8^*^	2.54	0.015
Ln*x*_6_	0.122 3^***^	6.35	<0.001
*cons*	−6.578^***^	−7.96	<0.001

^***^ indicates *p* < 0.001. ^**^ indicates *p* < 0.01. ^*^ indicates *p* < 0.05.

## 4 Discussion

### 4.1 Strengthening the spillover effect of the coordinated development of the eastern provinces and giving full play to the effect of radiation and demonstration

China’s allocation of health resources and economic development exhibit significant regional disparities (40). The coupling coordination relationship of the eastern provinces is better than that of the central and western provinces. In this regard, it is crucial to fully utilize the planning and guiding capabilities of policy tools. It is necessary to enhance the spatial spillover effect of the eastern provinces and form a situation in which the high-value areas of coupling coordination degree radiate and drive the development of the low-value areas. In terms of health resource allocation, it is imperative to break down local administrative barriers, strengthen central coordination and horizontal fiscal transfers, encourage resource linkages and sharing between eastern provinces and central and western provinces, actively promote pilot demonstration experiences, and give full play to the ability of eastern provinces to radiate across municipalities, provinces, and regions to push forward the process of equalization of healthcare (41, 42). In terms of economic development, on the one hand, it is necessary to strengthen the complementarity of production factors and industrial structure in the eastern, central, and western regions and to develop complementary regional economies to promote the vertical extension of the economy (43). On the other hand, due to differences in natural conditions, socioeconomic conditions, and economic policies, each region should develop regional economies of different types to promote horizontal integration of regional economies. The horizontal and vertical integration of the regional economy can achieve high-level coordination between the regional economy and the allocation of health resources (44).

### 4.2 Completing the shortcomings in the allocation of grassroots health resources and promoting the overall progress of provincial healthcare

The inefficient allocation of grassroots healthcare resources is an important factor that hinders the coordinated development of health resource allocation and the economy in eastern provinces (45, 46). There are some problems in the allocation of grassroots healthcare resources, such as redundancy, mismatch, and waste (47). In recent years, China’s medical reform policy has focused on building a grassroots healthcare service system, and the development of grassroots healthcare has good supporting conditions (48). To further optimize the allocation of health resources in provinces and promote higher-level coordination among systems, we put forward the following suggestions. First, we must make it clear that the development of grassroots healthcare has always been a weak link in the overall construction of the medical and health system (49). Second, at the grassroots level, the allocation of health resources should be based on the actual demand. Taking into account factors such as population, economy, and policies, adjusting the development scale according to local conditions, focusing on promoting the sinking of high-quality medical and health resources, strengthening the construction of a hierarchical diagnosis and treatment system, and improving the efficiency of health resource utilization are all effective measures (50, 51). Finally, the comprehensive evaluation system of grassroots healthcare resource allocation needs to be improved. The financing and distribution of health resources lack flexibility and standardization (52). Therefore, it is necessary to incorporate grassroots health resource allocation into “medical big data” for unified evaluation and standardized management. In addition, we need to strengthen information construction and digital management to fill the gaps in grassroots healthcare and to promote the overall progress of provincial healthcare.

### 4.3 Cultivating the kinetic energy of economic development and strengthening economic support capacity

The balance between supply and demand is the inherent requirement of high-quality development, and economic development originates from supply-push and demand-pull (53). From the perspective of supply and demand, the kinetic energy of economic development mainly includes consumption kinetic energy, investment kinetic energy, trade kinetic energy, institutional kinetic energy, structural kinetic energy, and factor kinetic energy (54). The current situation and driving factors of the coupling coordination relationship between health resource allocation and economic development have higher requirements for economic development momentum. In response, we provide the following suggestions to cultivate economic development momentum and strengthen economic support capacity. First, both the supply and demand sides should make efforts to adjust the regional macro economy to improve the system design of health resource allocation and economic development to inject institutional impetus into the optimal allocation of regional health resources and economic high-quality development (55). Second, we can guide and create new demand guided by structural adjustment and factor supply to promote the adjustment of industrial structure. By promoting resource flow to the health service industry, we will vigorously develop the health service industry to cultivate structural kinetic energy and factor kinetic energy to promote the coordinated development of the system (56). For example, exploring medical device product innovation can enrich factor supply. It is a two-way driver for achieving health resource allocation and economic development (57). Third, in terms of demand-driven economic development, China is making great efforts to build a long-term mechanism by capturing market demand to expand domestic demand and external demand to cultivate consumption, investment, and trade kinetic energy (58).

## 5 Conclusion

Based on the analysis above, we found that the allocation of health resources and the level of economic development in the eastern region of China had been improving year by year, and the system coupling coordination relationship had transitioned from low-level coordination to high-level coordination. We also concluded that health resource allocation and economic development are highly symbiotic, and industrial structure, investment in science and technology, and investment in health manpower and health material resources had significantly positive effects on the coordinated development of the system. Consequently, to achieve high-level coordination between health resource allocation and economic development in China, we propose to enhance the spillover effect of system coordination in the eastern provinces and fully compensate for the effect of radiation and demonstration to compensate for the shortcomings in the allocation of health resources at the grassroots level, boost the overall progress of provincial healthcare, cultivate the kinetic energy of economic development and strengthen the ability to support the economy.

Inevitably, our study has certain limitations. Firstly, in order to gain insight into high-quality development and high-level coordination in China, our study focused on the eastern provinces, which are characterized by a high level of economic development. As a result of this, our study failed to consider other regions and to make a comparison between the East and the Midwest. Secondly, the indicators included in our study were determined by reference to existing literature and expert recommendations, and the selection of indicators was limited by the database, which may have resulted in the omission of indicators. In future research, we will consider China’s systemic coordination and high-quality development from both national and international perspectives, and explore paths to enhance China’s systemic coordination and high-quality development through comparative analyses across regions and countries.

## Data Availability

Publicly available datasets were analyzed in this study. This data can be found at: https://www.stats.gov.cn/sj/ndsj/.
